# Fast simultaneous assessment of renal and liver function using polymethine dyes in animal models of chronic and acute organ injury

**DOI:** 10.1038/s41598-017-14987-5

**Published:** 2017-11-13

**Authors:** A. T. Press, M. J. Butans, T. P. Haider, C. Weber, S. Neugebauer, M. Kiehntopf, U. S. Schubert, M. G. Clemens, M. Bauer, A. Kortgen

**Affiliations:** 1 Department for Anesthesiology and Intensive Care Medicine, Jena University Hospital, Am Klinikum 1, 07747 Jena, Germany; 2 Center for Sepsis and Control and Care, Jena University Hospital, Am Klinikum 1, 07747 Jena, Germany; 3 Laboratory of Organic and Macromolecular Chemistry (IOMC), Friedrich Schiller University Jena, Humboldtstr. 10, 07743 Jena, Germany; 4 Jena Center for Soft Matter (JCSM), Friedrich Schiller University Jena, Philosophenweg 7, 07743 Jena, Germany; 5 Department for Clinical chemistry and Laboratory Medicine, Jena University Hospital, Am Klinikum 1, 07747 Jena, Germany; 6 Department of Biological Sciences and Center for Biomedical Engineering and Science, University of North Carolina at Charlotte, 9201 University City Blvd, Charlotte, NC 28223 USA

## Abstract

Simultaneous assessment of excretory liver and kidney function is still an unmet need in experimental stress models as well as in critical care. The aim of the study was to characterize two polymethine-dyes potentially suitable for this purpose *in vivo*. Plasma disappearance rate and elimination measurements of simultaneously injected fluorescent dyes DY-780 (hepato-biliary elimination) and DY-654(renal elimination) were conducted using catheter techniques and intravital microscopy in animals subjected to different organ injuries, i.e. polymicrobial sepsis by peritoneal contamination and infection, ischemia-reperfusion-injury and glycerol-induced acute kidney-injury. DY-780 and DY-654 showed organ specific and determined elimination routes in both healthy and diseased animals. They can be measured simultaneously using near-infrared imaging and spectrophotometry. Plasma-disappearance rates of DY-780 and DY-654 are superior to conventional biomarkers in indicating hepatic or kidney dysfunction in different animal models. Greatest impact on liver function was found in animals with polymicrobial sepsis whereas glomerular damage due to glycerol-induced kidney-injury had strongest impact on DY-654 elimination. We therefore conclude that hepatic elimination and renal filtration can be assessed in rodents measuring plasma-disappearance rates of both dyes. Further, assessment of organ dysfunction by polymethine dyes correlates with, but outperforms conventional biomarkers regarding sensitivity and the option of spatial resolution if biophotonic strategies are applied. Polymethine-dye clearance thereby allows sensitive point-of-care assessment of both organ functions simultaneously.

## Introduction

(Multi-)organ dysfunction after systemic infection defines sepsis^1^ and is one of the most common causes of death in intensive care units worldwide. The lack of treatment options has stimulated extensive translational research in rodent models in which a reliable assessment of organ dysfunction *in vivo* is critical. Most of the treatments focus on temporary organ support or replacement therapies, e.g. inotropic and vasopressor support in cardiovascular, dialysis in kidney or mechanical ventilation in respiratory failure. As no specific treatments are available to restore individual organ functions, preventive and early supportive therapeutic treatment modalities are of particular importance in critical care. Timely diagnosis of impaired organ function is crucial in this context.

Kidney and liver dysfunction are of particular importance, since both contribute to exacerbated remote organ impairment due to accumulation of endo- and xenobiotic substances. However, diagnostic tools for sensitive and rapid detection of organ dysfunction for kidney or liver failure in daily clinical practice are still scarce. Most biomarkers currently in use are static plasma or urine parameters such as bilirubin in case of liver dysfunction or creatinine and urea for kidney failure. These parameters, however, often lack sensitivity and specificity. E.g. due to the high storage capacity of the liver an increase in bilirubin is a rather late event. Therefore, so called dynamic markers such as the plasma disappearance rate of indocyanine green (ICG) or the ^13^C-methacetin breath test (LiMAx) have been investigated to better assess liver function in critical care patients. ICG is an infrared fluorescent polymethine dye which is eliminated exclusively via the hepato-biliary route without further metabolism, while methacetin is metabolized by CYP 1A2. Particularly, PDR_ICG_ was found to be a sensitive marker for liver function and is well documented to detect liver dysfunction^2–4^ earlier than plasma markers, such as bilirubin^5^. Assessing acute kidney injury early in the critically ill patient is still a great challenge and may be of substantial importance to improve prognosis by earlier and, therefore more effective, treatment. In addition to plasma urea and creatinine levels, the albumin-creatinine ratio to detect microproteinuria and inulin-clearance to measure kidney function (glomerular filtration rate) are also used. However, these parameters lack sensitivity^6^ or for inulin-clearance require urine sampling up to 24 h making timely diagnosis impossible. Hence, these methods might not be sufficient for timely assessment of glomerular filtration rate^7^. There is an ongoing search for better diagnostics and new biomarkers such as up-regulated proteins, e.g. neutrophil gelatinase-associated lipocalin (NGAL)^7–9^, urinary kidney injury molecule (KIM-1)^10–12^, insulin-like growth factor-binding protein 7 (IGFBP7) and tissue inhibitor of metalloproteinases-2 (TIMP-2)^13–15^. Enzymes measured, such as NAG, a- and p-glutathione s-transferase (GST), γ-glutanyl transpeptidase (γ-GT) and alkaline phosphatase (AP) as well as the functional marker plasma/serum Cystatin C have limited clinical utility:^16^ These markers cannot be applied at point-of-care, when immediate assessment of the effect of a treatment is needed. To conclude, a fast, sensitive method to monitor renal function easily in a point-of-care fashion in the critically ill is not available and represents a bottleneck in acute care. To address the limitations of simultaneous assessment of excretion by liver and kidney we developed a system based on two infrared polymethine dyes. Their distinct spectral properties allow to simultaneously analyze liver and renal dysfunction *in vivo* applying plasma-disappearance rate (PDR) measurement in animal models for liver and kidney ischemia-reperfusion (IR) injury, glycerol-induced acute kidney injury (AKI) and a model for sepsis, i.e. peritoneal contamination and infection, which can lead to multiple organ dysfunction and would allow to study the organ-organ interactions critical for the pathophysiology of acute care.

## Results

**Figure 1:**
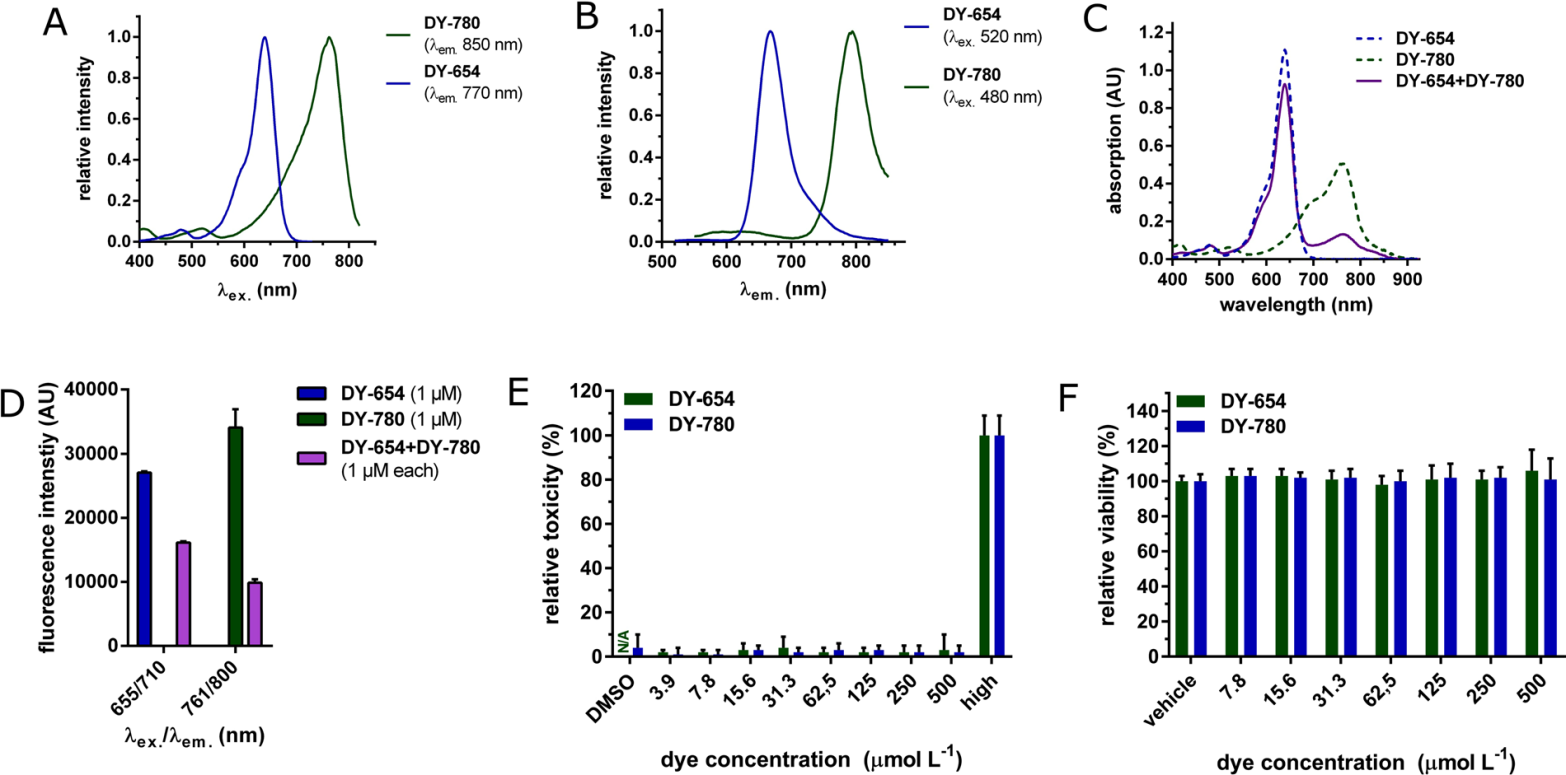
(Near)infrared polymethine dyes for *in vivo* use. (**A**) Excitation and (**B**) Emission spectra of DY-654 and DY-780 in PBS, 0.1% DMSO. λ_em_ or λ_ex_ in (**A**) and (**B**) refer to the wavelength at which the dyes were excited to measure emission spectrum or the wavelength at which emission was measured while measuring excitation spectrum. (**C**) Absorption spectra of unmixed and equimolar mixed dyes. (**D**) Specific excitation and emission wavelengths allow the simultaneous detection of both dyes in whole blood. (**E**) Toxicity (LDH-assay) after 24 h on primary murine hepatocytes for DY-654 and DY-780 was assessed revealing no cellular toxicity. LDH release is expressed in % to completely lysed cells (High) by Triton-X solution. DY-780 was incubated in 0.005% DMSO, therefore a vehicle-control (DMSO) was included (**F**) The alamar blue assay revealed no change in cell viability of primary murine hepatocytes after incubation with DY-654, DY-780 or vehicle (medium or DMSO) for 24 h (**E,F**) Hepatocytes from 10 (LDH-Assay) or 6 (Alamar Blue Assay) different animals were used for the experiment.

Polymethine dyes are described to have organ specific elimination routes^17^. Also ICG is already approved as a clinical test to analyze liver function. In this study we used two polymethine dyes with distinct elimination routes and fluorescence spectra (Fig. 1). DY-654, a near infrared-dye with four sulfonic residues, is excreted via the kidney and DY-780, which has a quite similar fluorescence spectrum to ICG, is excreted via the hepato-biliary route. The low molecular mass of the dyes used (739.42 g mol^−1^ for DY-780 and 1027.18 g mol^−1^ for DY-654) would allow rapid glomerular filtration. Absorption characteristics of DY-780 are altered significantly in the presence of serum in a similar manner as the hepatobiliary-cleared dye ICG. Interestingly spectral characteristics of DY-654 as well as the one of IRDye800cw, a dye known to be filtered by the kidney, did not change in the presence of serum (Fig. S1A). Further the presence of an interaction of the dyes with albumin can be demonstrated by dialysis of the different dye-solutions against buffer in the presence and absence of 15% of albumin. The presence of albumin reduced the diffusion of DY-780 and ICG through a dialysis membrane (molecular mass cut off: 6000 to 8000 Da) but not DY-654 and IRDye800cw (Fig. S1B) suggesting that albumin binding of DY-780 would impede filtration by the kidney while the unbound DY-654 is freely filtered. The peak maxima of both dyes differ by 129 nm in the excitation spectra (Fig. 1A) and by 123 nm in the emission spectra (Fig. 1B) in phosphate buffered saline (Biochrom AG, Germany) (PBS). Equimolar mixing of both dyes does not alter the distinct absorption peaks (Fig. 1C) and enables measurement of both dyes without spillover (Fig. 1D). The spectra further allow one to choose from a variety of different microscopic and (near)infrared imaging methods to analyze non-invasive plasma-disappearance rate. Fluorescence intensity loss due to photo-bleaching in an aqueous physiologic buffer (Krebs-Henseleit Buffer, KHB) was negligible (0.7%/100 cycles for DY-654, 3.7%/100 cycles for DY-780, 100 flashes per cycle) (Fig. S1C). The addition of 5% bovine serum albumin to KHB, further reduced signal loss to (0.09% and 0.18% per 100 cycles for DY-654 and DY-780 respectively) (Fig. S1D). DY-780 and DY-654 did not lead to cellular toxicity or decrease viability of primary murine hepatocytes in concentrations between 3.9 to 500 μmol L^−1^ in (Fig. 1E,F). Cellular injury in two cell lines (HepG2 (for DY-780) and HEK293 (for DY-654)) was also not detectable by LDH-assay (Fig. S2A,B).

However DY-780 slightly compromises viability, measured using alamar blue assay, (Fig. S2C) which reaches significance for DY-654 at high concentrations after 24 h, c(DY-654) ≥50 µM and 72 h for c(DY-654) ≥1 µM (Fig. S2D).

**Figure 2:**
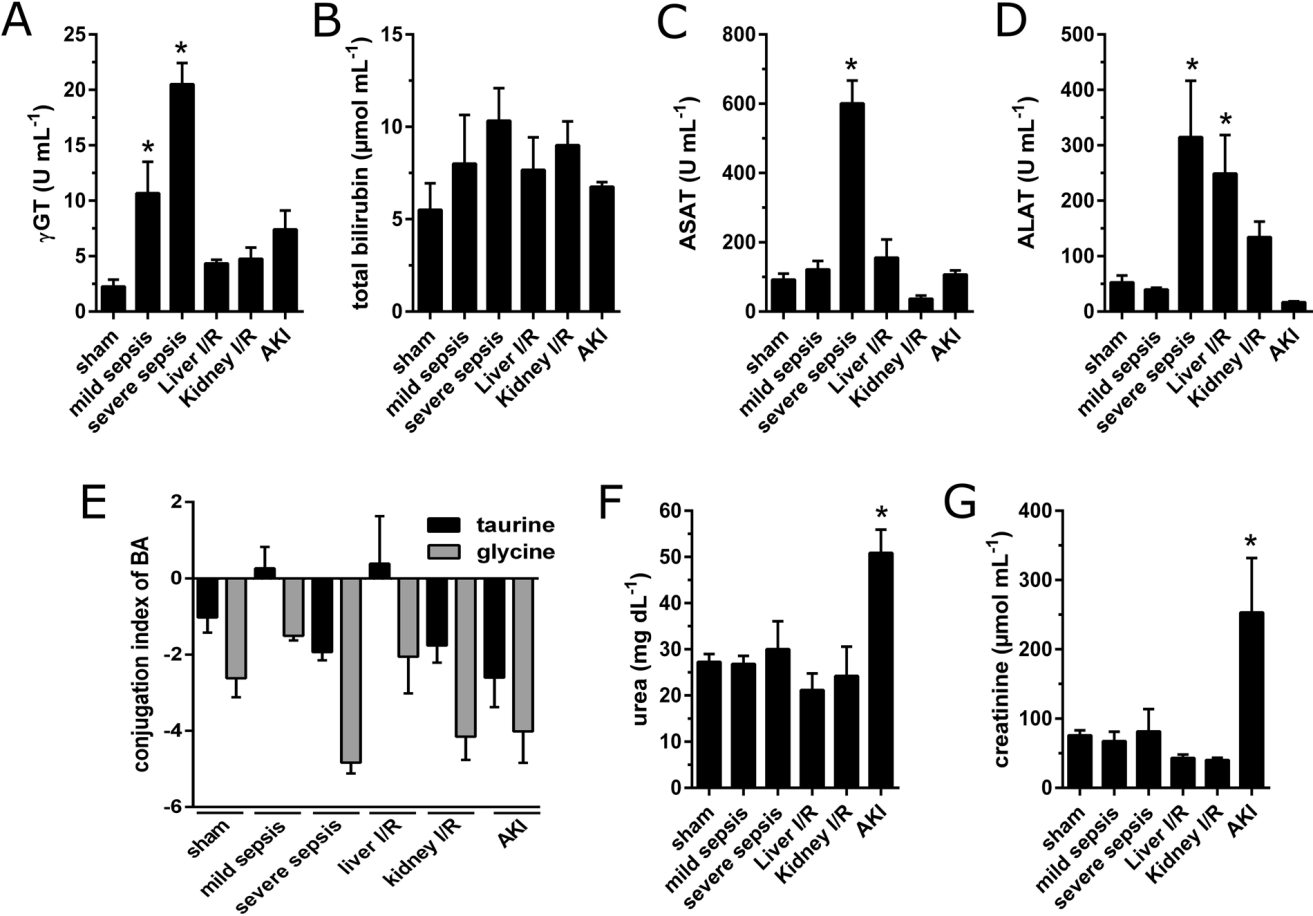
Conventional biomarkers of organ damage. Biomarkers indicating liver damage in sepsis (n = 3 for mild sepsis, n = 4 for severe sepsis) and liver ischemia-reperfusion injury (n = 4): total bilirubin (**A**), γ-GT (**B**), as well as transaminases ASAT (**C**) and ALAT (**D**). Conjugation index shows the log_2_-transformed ratio of the sum of taurine- or glycine-conjugated BA in lithium-heparin plasma and the sum of the related unconjugated species (**E**). Kidney damage by AKI (n = 3) but not kidney-IR (n = 4) lead to a significant increase in plasma urea (**F**) and creatinine (**G**). *Depicts significance, Friedman Test post-hoc Dunn’s test against sham-group; α < 0.05.

Besides image-based diagnostics, the measurement of surrogate parameters is an important factor to diagnose and analyze disease progression. To monitor liver function, ICG-clearance can be utilized but plasma bilirubin level is used most frequently,e.g. scoring systems, such as the Sepsis-related organ failure assessment (SOFA) score^1^, primarily because of its widespread availability. However, as also shown in Fig. 2A, plasma bilirubin is an insensitive biomarker for liver function^18^ since hepatocytes have a great storage capacity^9^.

Thus neither animals with severe sepsis nor liver injury show a significant functional impairment as assessed by plasma bilirubin levels (Fig. 2A). However plasma-markers γ-GT (Fig. 2B) and transaminases (Fig. 2C,D) revealed liver damage in those models but do not measure “liver function”^19^. Another possibility to assess liver function is to track the hepatic conjugated bile acids (BA). Therefore the log_2_-transformed ratio of the sum of plasma-taurine or -glycine conjugated bile acids to their unconjugated precursors were calculated as we have described earlier^5^. Animals classified with mild sepsis or liver IR injury had a significant accumulation of taurine-conjugated BA reflecting an excretory dysfunction, whereas animals with severe sepsis showed a slight decrease of taurine- and glycine-conjugated BA due to an impaired phase 1 and 2 biotransformation (Fig. 2E). Renal damage due to IR or acute kidney dysfunction was assessed by plasma urea (Fig. 2F) and creatinine (Fig. 2G). Neither marker detected kidney dysfunction after IR injury, but both did after induction of AKI.

**Figure 3:**
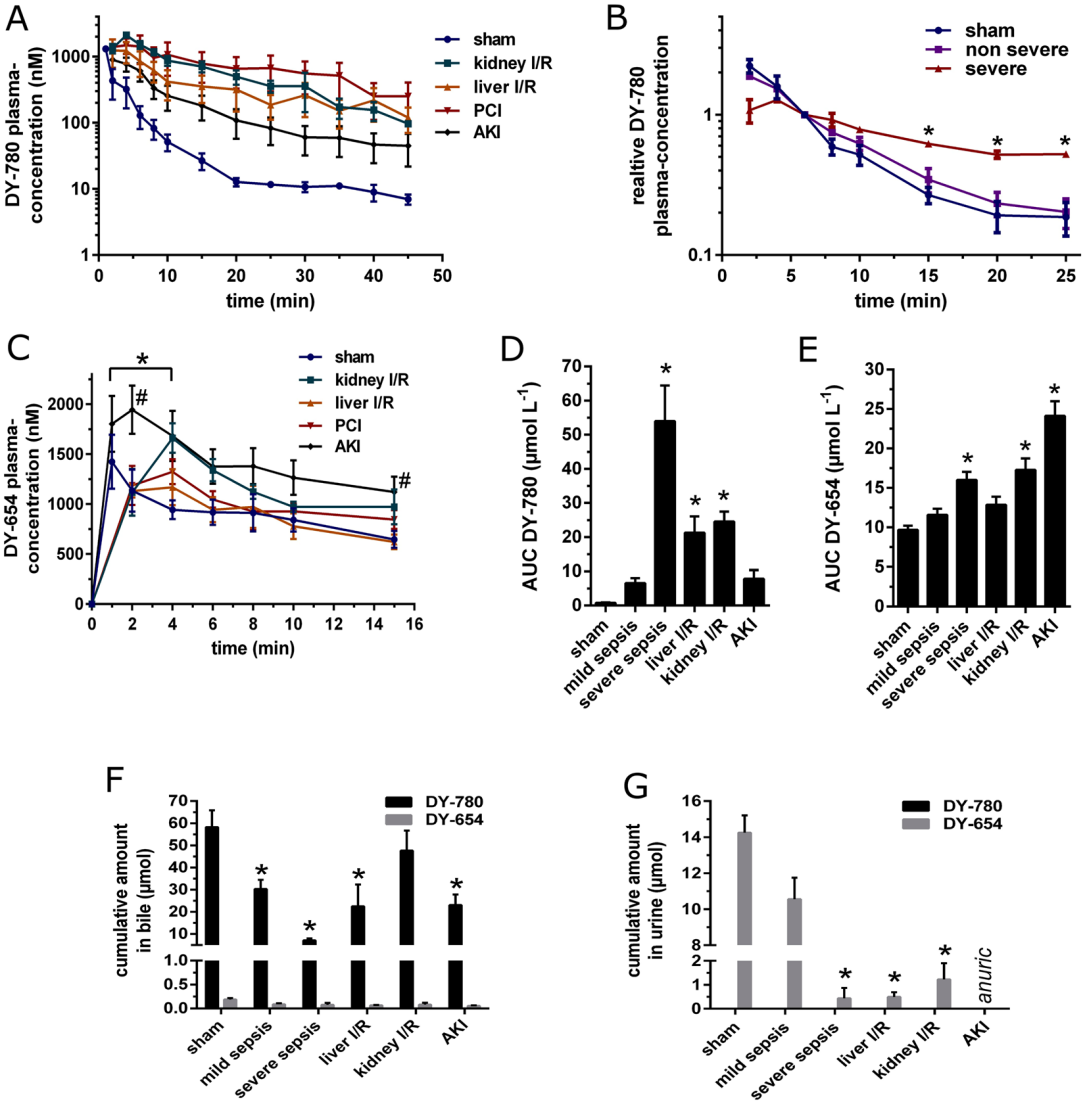
Polymethine dyes to assess liver and kidney function simultaneously. Hepato-biliary plasma clearance of DY-780 (**A, B**). * in (**B**) depicts significance of DY-780 plasma concentration between severe sepsis (n = 4) and sham (n = 4). Renal cleared DY-654 in different rodent animal models (**C**): DY-654 plasma concentration reaches an increased peak in animals with acute renal dysfunction after i.m. glycerol injection (n = 3) compared to sham (#). Other animal models (kidney (n = 4) or liver IR (n = 4) injury and PCI (n = 7)) lead to a significant delayed plasma peak (*). AUC analysis showed a significant increase in case of functional liver (**D**) or renal impairment (**E**). (**F**): Biliary excretion of DY-780 is significantly lowered in PCI, liver IR and AKI, but not in kidney IR compared to sham animals (*). No significant hepato-biliary excretion of DY-654 was found in all groups. (**G**): Renal excretion of DY-654 was significantly lowered in liver and kidney IR as well as severe sepsis compared to sham (*). The AKI model lead to anuria in rats thus no urine could be sampled. Further no DY-780 was found in urine. (**B** to **G**): # and *, α < 0.05, Friedman Test post-hoc Dunn’s test.

Besides measuring different established biomarkers in the different animal models, the PDR of simultaneously i.v. administered DY-780 and DY-654 (140 pmol g^−1^ BW each) was measured, to assess kidney and liver function, respectively. DY-780 showed prolonged elevated plasma concentration in all animal models of organ damage (Fig. 3A). Interestingly peritoneal contamination and infection (PCI) animals, classified using transaminases as markers of mild vs. severe liver damage, showed significantly different kinetics (Fig. 3B). DY-654 PDR showed a significantly delayed and slightly flattened plasma peak compared to control in sham-treated animals. Subsequent to AKI the plasma peak was significantly increased (Fig. 3C). As shown in Fig. 3D for DY-780 and Fig. 3E for DY-654 these alterations in the PDR can be quantified calculating the area under the curve (AUC) as a marker for organ function, where an increased AUC indicates organ dysfunction. In line with these findings biliary excretion of DY-780 was significantly reduced in PCI, liver IR and AKI (Fig. 3F). Renal excretion of DY-654 was reduced in all models associated with kidney injury (Fig. 3G). In healthy animals the DY-780 recovery in the bile within one hour was 84.2% ± 12.4% and for DY-654 in the urine 76.8% ± 8.6% (both mean ± s.d.). Additionally the data confirm that there is no excretion of DY-780 via the kidneys or DY-654 through the hepato-biliary route respectively; thus PDRs are not altered due to compensatory excretion of a dye through other than the primary route in all injury models tested (Fig. 3F,G).

**Figure 4:**
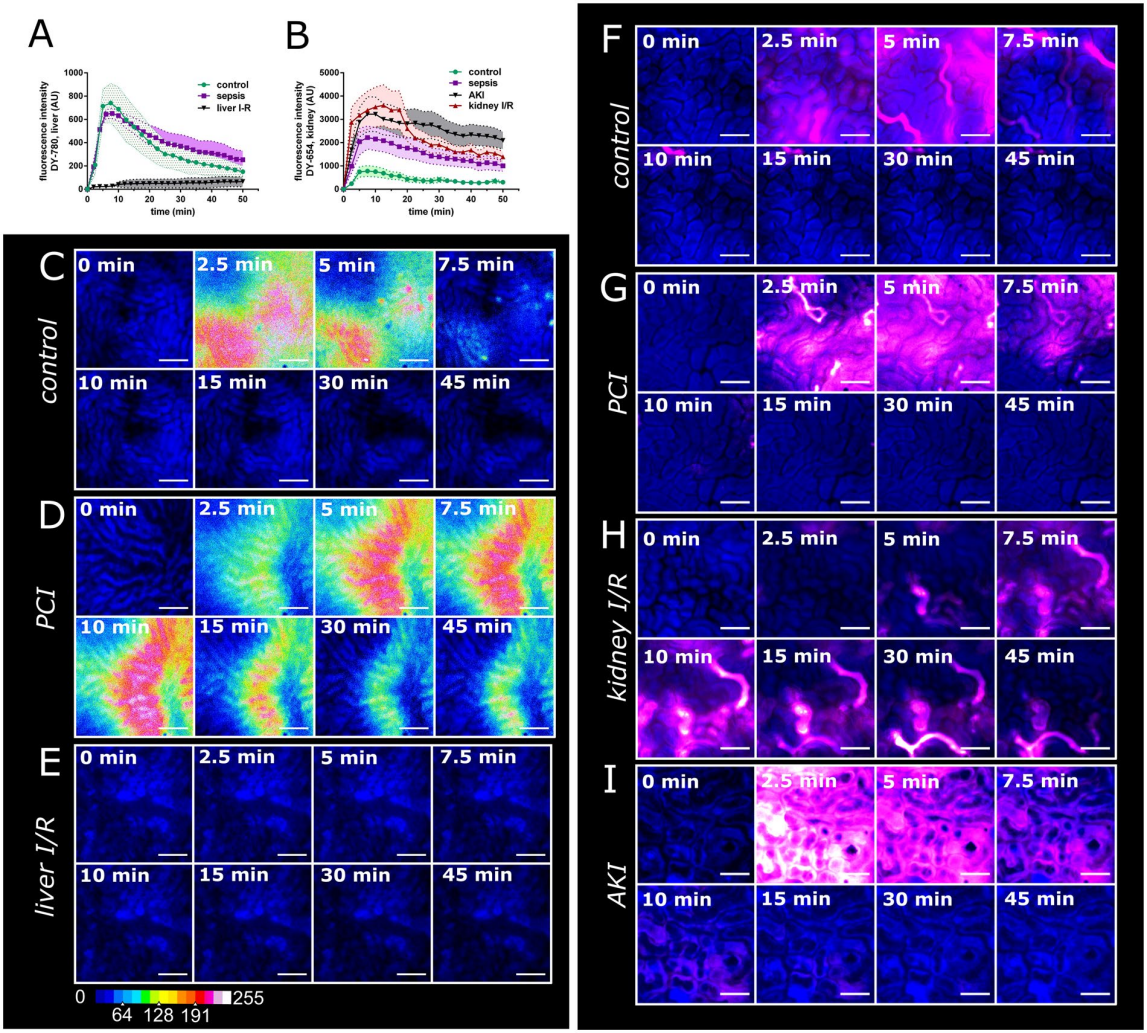
Intravital microscopy of DY-654 and DY-780 in different animal models associated with organ damage. Images and kinetics obtained via epifluorescence IVM of DY-780 in rat liver or DY-654 in rat kidney, respectively. Fluorescent decay of DY-780 in liver tissue (**A**) and DY-654 in kidney tissue (**B**). Images in the left panel show different distribution pattern of DY-780 (16 color heat map) in control (**C**), PCI (**D**) and liver IR (**E**) over time. The right panel depicts DY-654 (magenta) distribution in control (**F**), PCI (**G**), kidney IR injury (**H**) and animals with AKI (**I**). Scale bar = 200 μm (**C** to **E**), 100 μm (**F** to **I**). NAD(P)H autofluorescence is exploited to visualize tissue (blue). Minimum 4 areas of the liver or kidney were measured simultaneously in 3 independent experiments per group.

The validity of PDR monitoring was further illustrated and confirmed by intravital microscopy. The fluorescence decay of DY-780 or DY-654 follows a kinetic of 1^st^ order in the case of healthy animals or animals with mild sepsis. This kinetic was significantly delayed under severely septic conditions, which is in line with previous intravital microscopy studies of ICG (Fig. 4A)^20,21^. Images of DY-780 in hepatic tissue shows a zone 1 uptake of the dye in healthy animals after 10 min with rapid signal decay over time (Fig. 4C). In contrast animals that underwent PCI treatment showed an increased uptake in zone 3 of the liver and slow signal decay (Fig. 4D). Animals that underwent liver IR suffered from hepatic circulation failure. Only little if any dye was transported to the subcapsular liver surface within 50 min (Fig. 4A) resulting in no visible signal in the tissue (Fig. 4E). The hypo-perfusion of the liver in this model is further indicated by the low metabolic activity of the tissue indicated by the low nicotinamide adenine dinucleotide (phosphate) (NAD(P)H) autofluorescence (Fig. 4E). Kidney injury leads to differences in maximal fluorescence intensity values of DY-654 but constant signal decay (0 order kinetic) in renal tissue between control, PCI, kidney IR and AKI. Further control animals had the lowest maximal plasma concentration followed by PCI and AKI suggesting increased DY-654 accumulation within increase of kidney damage. Kidney IR resulted in a delayed increase in fluorescence intensity as well as highest plasma concentrations. In contrast to the other injury models the maximum plasma-concentrations were followed by an exponential decay (1^st^ order kinetic) and a significantly delayed kinetic was observed in the other animal models (Fig. 4B).

The images revealed a quick appearance in different tubular structures at different time-points (Fig. 4F, Suppl. Video 1) leading to the conclusion that DY-654 is actually filtered by the kidney via the glomerular system rather than secreted through tubular cells. Both substances DY-654 and the polymer-conjugate accumulated only in the tubular system and might be even partly reabsorbed there by the tubular cells, however active uptake or interaction of DY-654 was not found in any experiments (Fig. S3C,D and Video S3) leading to the conclusion that DY-654 is filtered by glomeruli. The tubular system further is more globally stained with DY-654 in animals 16 h after PCI (Fig. 4G). As already depicted by the fluorescence intensity analysis, kidney IR led to a slightly delayed accumulation of DY-654 in the tubular system which is very prominent in some tubular segments during the whole measurement (Fig. 4H). AKI finally resulted in an early and diffuse staining of all tubular segments, vessels and cellular structures. In contrast to all other animal models, in AKI tubular cells remained DY-654 positive after a few minutes whereas the tubular lumen is hardly stained (Fig. 4I).

**Figure 5:**
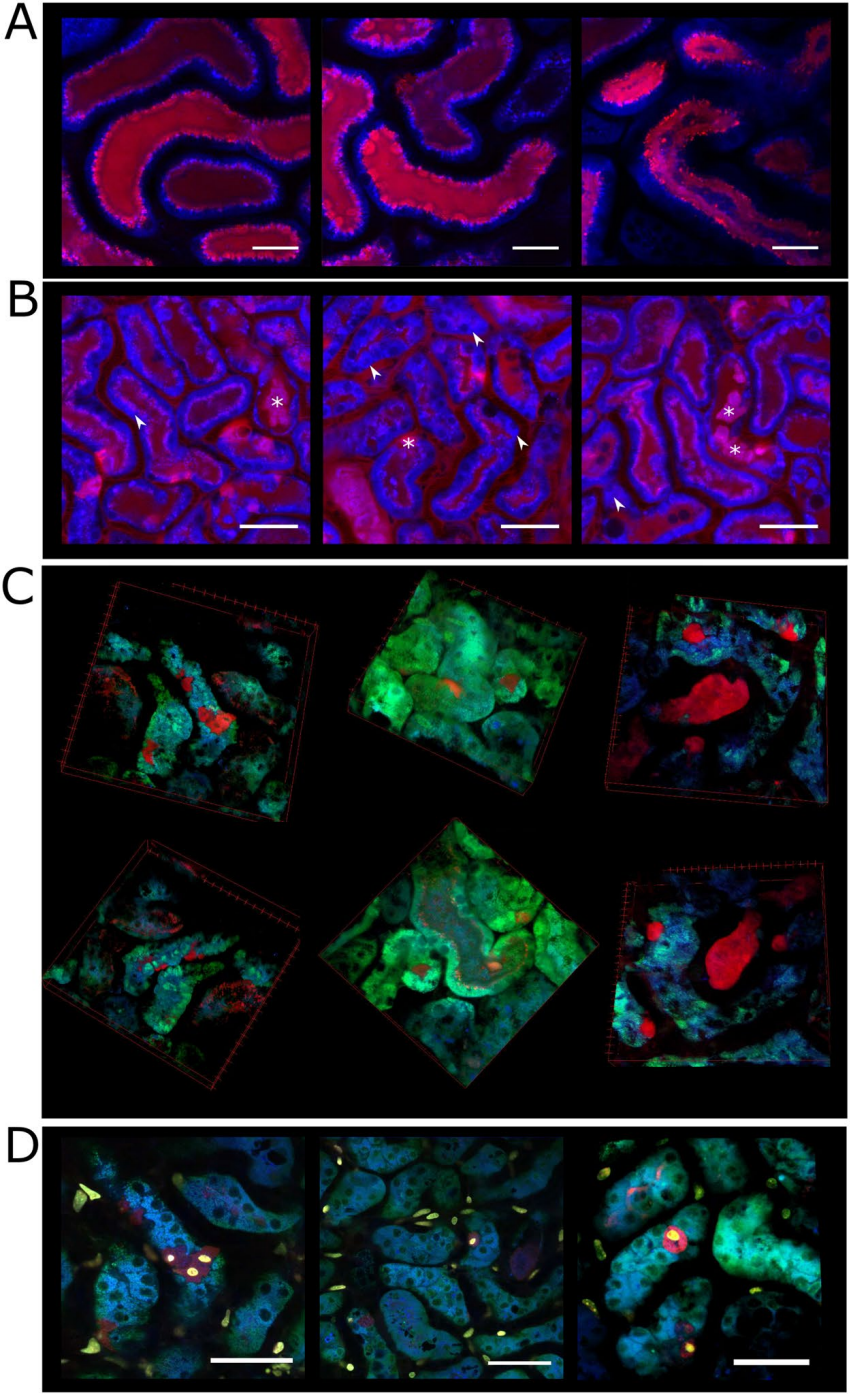
Microscopic analysis of DY-654 distribution in murine kidneys. Representative images from confocal IVM of DY-654 (red) in control (**A**) animals and after induction of AKI (**B** to **D**). **(B)**: Asterisks point out some cellular debris in the tubular wall or lumen. Arrowheads point towards focal increase of NAD(P)H autofluorescence depicting tubular damage. (**C**): 3D-reconstruction of renal tubular system 30 min after DY-654 injection. The mitochondrial staining MitoDY-1 (green) was administrated 12 min before image acquisition. (**D**): Images of DY-654 90 min after administration. Additionally dead cells were stained 10 min before image acquisition using intravenous PI (yellow) administration. DY-654 only accumulates in PI positive cells. NAD(P)H autofluorescence is exploited to visualize tissue (blue). Scale bar = 100 μm (**A**), 50 μm (**B,D**). All experiments were carried out at least three times.

To test, whether DY-654 is filtered, we conjugated the dye covalently to PoEtOxMA, a high molar mass polymer. This comb-shaped polymer is stretched in aqueous solution because the steric hindrance induced by the side chains prevents a coiling of the polymer backbone (Fig. S3A)^22^. Thus, the conjugate resembles a cylindrical shape under physiological conditions exposing the DY-654 at one end. Based on earlier detailed investigations of this polymer type and the absolute molar mass of the sample, the cylinder is estimated to be of 15 nm in length and 4 nm in diameter^22^. Thereby filtration was significantly retarded compared to free DY-654 (Fig. S3A,B) and still no interaction with basolateral tubular membrane occurred (Fig. S3C,D). It should be noted that all polymer-dye conjugates contain either one or none label per polymer chain, while the possibility of several dye molecules attached to the same polymer chain is excluded by the choice of the synthetic pathway, described in detail in the Supplementary information and Supplementary Scheme 1 as well as Figure S4 and S5. The cellular uptake of DY-654 in animals with AKI was surprising considering all data suggested a filtration of the dye. Thus, confocal intravital microscopy was applied to analyze the distribution further (Fig. 5). Mice injected with DY-654 showed a fast appearance in different tubular sections. At higher magnifications DY-654 localizes in the lumen with some associations towards the inner tubular membrane, showing the formation of apical tubular blebs^23^ at early time points (Fig. 5A, Suppl. Video 1). This pattern is strongly altered in mice with AKI. Besides a prolonged intravascular phase of DY-654, the tubular lumen is only weakly stained compared to control mice. Further some NAD(P)H and DY-654 positive cell debris can be found in the lumen (Fig. 5B, asterisks). In addition, several tubular walls are strongly DY-654 positive giving an impression DY-654 is leaking through these cells in the lumen. The severe cellular damage caused in this model is additionally indicated by the strong focal NAD(P)H autofluorescence of the tubular cells (Fig. 5B, arrowheads; Suppl. Video 2)^24^. In line with the inhomogeneous NAD(P)H intensity distribution mitochondrial staining using MitoDY-1 revealed similar cellular staining patterns *in vivo*. The 3D-reconstruction shows single cells or small areas which are DY-654 positive and partly detached from their cell-group (Fig. 5C). Additional i.v. administered propidium iodide (PI) accumulates in nuclei of apoptotic and necrotic cells identifying DY-654 positive cells as such (Fig. 5D).

## Discussion

In this study we demonstrated feasibility of simultaneous assessment of hepatic and renal function in different rodent models of liver and kidney impairment using two polymethine dyes with distinct elimination routes.

Multiple organ dysfunction represents a major complication and may lead to a vicious cycle in critical illness, e.g. sepsis. While supportive therapies offer a partial and temporary means to bridge patients until recovery, no specific treatment is currently available to induce recovery of organ functions. Liver failure has a particularly grave outcome in critically ill patients since no permanent replacement therapy is available. Liver function replacement therapy can only partially replace the liver in critical functions such as ammonia clearance, detoxification and clotting factor synthesis, and only for a limited time, to support regeneration or until liver transplantation takes place. It has been demonstrated that measuring PDR-ICG using pulse dye densitometry^25^ or the recently described multispectral optoacoustic tomography enables sensitive and rapid assessment of liver function^26,27^. Estimation of glomerular filtration rate via inulin-clearance measurement represents today’s gold standard to assess kidney function but this requires time consuming urine sampling and fails to assess rapid changes in organ function prototypical for the intensive care unit. Other techniques such as technetium-99m diethylene triamine penta-acetic acid clearance are also used to evaluate kidney function with respect to filtration^28–30^. However, these methods are time-consuming to conduct and therefore very seldom used in intensive care units. More often measured is serum creatinine as well as the creatinine clearance, however, their accuracy and value in acute kidney injury is limited^31^. The use of radioactive tracer molecules needs a high patient compliance, and safety concerns make such radioactive techniques inapplicable for the daily measurements in an intensive care unit routine. Inulin or sinistrin covalently bound to fluorescein isothiocyanate (FITC) allows clearance measurements in experimental setups such as intravital microscopy or repetitive blood sampling^32–36^. However, since the absorption of FITC is overlaid by the one of hemoglobin, bedside use via pulse dye densitometry cannot be applied^33^. As a result, assessing renal dysfunction as quickly and simply as PDR-ICG allows for the liver is an unmet medical need. ICG as well as DY-780 and DY-654 are polymethine dyes, since they all possess a conjugated carbon-chain, referred to as polymethine, the length of the polymethine chain modulates their optical properties. These dyes have been shown to be eliminated through hepato-biliary route or kidneys dependent on their amount of sulfonic residues^17,37^. Hepato-biliary elimination of polymethine dyes and other compounds is the consequence of uptake and excretion. Hepatic uptake is affected by basolateral organic anion and cation transporters as well as the sodium-taurocholate co-transporting polypeptide^37,38^. Subsequent canalicular excretion takes place trough multi-drug resistance-associated proteins^39^. In this study ICG was replaced with the spectrally similar and more photo-stable DY-780 which has a distinct hepatic elimination. DY-780 was combined with DY-654, a hydrophilic dye with four sulfonic residues, to measure kidney function. Their optical properties allow simultaneous measurement of DY-780 and DY-654 without the need of linear unmixing or other compensatory algorithms. Additionally, both dyes absorb and emit in the optical window of blood bordered by the absorption peaks of (oxygenated) hemoglobin and water^40–42^. These optical properties make DY-780 and DY-654 suitable for monitoring by pulse dye densitometry and multispectral optoacustic tomography (MSOT). Our data show that DY-654 is filtered by the kidney and has only a negligible hepato-biliary secretion. We conclude that changes seen in PDR of DY-654 are due to impaired filtration but not tubular secretion. Notable, monitoring of DY-654 plasma-clearance for only 15 min can clearly indicate renal dysfunction. The superior sensitivity of this method is indicated since common biomarkers used for the evaluation of kidney function in critically ill patients did not indicate kidney injury in two of three models used. Only a severe glomerular damage associated with acute kidney failure induced by glycerol-induced rhabdomyolysis^43,44^ led to an increase in plasma-urea and creatinine concentrations. Concomitantly, this severe glomerular damage models also resulted in an elevated DY-654 plasma-peak concentration. Alterations in renal vascular resistance in the model of glycerol-induced acute kidney injury were described previously^45^. For this model, confocal intravital microscopy further revealed occurrence of cellular debris in tubular lumen and necrotic tubular cells, identified as DY-654 and PI positive, as well as necrotic tubular endothelial cells (DY-654 negative and PI positive), thus providing additional spatial and cell-type specific information regarding occurrence of injury and dysfunction.

Liver function assessed by DY-780 plasma clearance correlated with parameters of cholestasis and liver damage, i.e. γ-GT, aspartate aminotransferase (ASAT) and alanine aminotransferase (ALAT), while bilirubin levels were not significantly altered in tested groups. In addition, kidney IR led to a slight increase in total bilirubin, ASAT and ALAT. This was associated with liver dysfunction as indicated by PDR of DY-780 to a similar degree as in liver IR injury demonstrating remote organ dysfunction of the liver concomitant with renal dysfunction, rendering organ-organ interactions in multi-organ dysfunction syndroms accessible to real-time monitoring. Finally, (failing) conjugation of bile acids also indicated liver dysfunction as previously described^45^ and correlated with biophotonic assessment of excretory failure in models of mild sepsis and liver IR. Similarly, elevation of unconjugated bile acids in plasma indicates an intrahepatic failure of phase I and II biotransformation in severe sepsis and kidney IR.

In conclusion, measuring PDRs of the two polymethine dyes DY-654 and DY-780 allows simultaneous evaluation of hepatic and renal function. By applying techniques such as pulse-densitometry or multispectral optoacustic tomography^37^ this approach will, in principle, allow point-of-care diagnostics in critically ill patients and translational models used to unravel novel treatment options and organ-organ interactions in the acute care setting where conventional plasma markers are of limited utility to assess the kinetics of organ impairment.

## Materials and Methods

### Animals

All experimental procedures on animals were approved by the local government authority of Thuringia, *Thüringer Landesverwaltungsamt*, and carried out in accordance with the approved guidelines. Animals were housed under specific pathogen-free conditions in the animal facility of the Jena University Hospital. In general during all procedures and imaging methods, animals remained under deep general anesthesia using Isoflurane and pain-reflexes were assessed to gauge the depth of anesthesia. All surgical interventions were performed in a semi-sterile environment using tip-sterilized surgical instruments, and sterile equipment such as sutures, pads, gloves. Specific details of experimental methods are provided below with the description of the different models used.

### Preparation and characterization of polymethine dyes

DY-780 and DY-654 (both amino-terminated) (Dyomics GmbH, Germany) were dissolved separately in PBS containing 0.1% DMSO to 1 mmol L^−1^. Dyes were then diluted in DMEM/F12 (Biochrom, Germany) when used in cell culture. Fluorescence, excitation, and emission spectra of 1 µmol L^−1^ dye solutions were obtained using a multiplate reader (Tecan infinite 200 M, Tecan). For some assays and animal experiments dyes were mixed 1:1 (v/v) and further diluted in saline or whole blood from mice. Absorption spectra (2 nm resolution) of 5 µmol L^−1^ dye solution were obtained using a spectrophotometer (Multiskan GO, Thermo Scientific). DY-654 and DY-780 from samples were measured and quantified in urine, bile and plasma using standard curves prepared in the respective body fluid. Fluorescence intensities were analyzed using the Tecan plate reader (DY-780: λ_ex._: 776 ± 9 nm; λ_em._: 815 ± 0). In experiments with amino-modified DY-654 or DY-780, Indocyanine green (Pulsion, Germany) or IRDye800cw carboxylate (Licor Inc., USA) dyes were diluted to 1 or 5 µmol L^−1^ in Krebs-Henseleit Buffer (KHB) (Biochrom, Germany). For some experiments up to 25% bovine serum albumin fraction V (BSA) (Serva Electrophoresis GmbH, Germany) was added. 1 mL of the dissolved dye was dialyzed twice against 200 ml KHB for 8 h each time using a Spectra/Por Dialysis membrane (Spectrum Labs, Germany) with a molecular weight cut off of 6000 to 8000 Da. Fluorescence of dialyzed solution was measured before and after the experiment using the Tecan plate reader using the settings described above. For ICG and IRDye800cw the same detection settings as for DY-780 were used. Data were quantified using standard curves of the dyes in the KHB with or without albumin and fraction of dye permeating the dialysis tubing was calculated. Photostability was assessed by diluting the dyes in KHB or KHB containing 5% BSA to 0.1 µmol L^−1^ in black 96 well-plates. The wells were excited 500 times (each time, 100 flashes, approximately 2.5 s in-between every cycle) at 37 °C (EnSpire Multimode Plate Reader, PerkinElmer). The fluorescence intensity of the first measurement (mean of 100 flashes) was defined as 1 and the change of fluorescence intensity for each repetition was expressed as fraction to the first measurement. The experiment was carried out three times. Calculated signal changes refer to the relative signal loss during 100 exposures which was calculated from the linear regressions using the mean values from the replicates.

### Hepatocyte Isolation

Hepatocytes were isolated from adult C57BL/6 J mice using a standard collagenase perfusion method. Mice were sacrificed by an overdose isoflurane. After the death of the animal was confirmed a laparotomy was performed and the portal vein cannulated with a 25 G needle connected to a perfusion setup. The liver was perfused at 6.5 ml min^−1^. An outlet for the blood and buffer was created by cutting the descending aorta right after the cannula was inserted. All buffers were sterile filtered (0.45 µm PVDF membrane), fully oxygenated with carbogen gas, at pH 7.4 at a temperature of 37 °C (measured at the outlet of the 25 G needle). The liver was first perfused for approximately 5 min with PBS containing 1.98 g L^−1^ glucose, 5 mmol L^−1^ HEPES followed by perfusion with 20 to 28 mg collagenase Type IV (Worthington Bioscience), > 160 U/ mg dry weight) (0.5 mg m L^−1^, solved in the PBS w/o Ca^2+^, w/o Mg^2+^, 1.98 g L^−1^ glucose, 5 mmol L^−1^ HEPES, 5 mmol L^−1^ CaCl_2_). Afterwards the liver was excised, the gall bladder removed and the hepatocytes emptied in DMEM (4.5 g mL^−1^ glucose) (Biochrom, Germany) containing 100 IU mL^−1^ penicillin, 100 IU mL^−1^ streptomycin and 10% fetal calf serum (Thermo Scientific, Germany). Hepatocytes were subsequently filtered through a 100 µm cell strainer and collected and purified by centrifugation at 50 x g, 3 min for 3 times with exchanged medium. Cell viability was assessed afterwards by trypan blue staining, indicating viability > 93%. Nonparenchymal cells were not detected by flow cytometry. Hepatocytes were seeded (0.05 × 10^6^ per well) on collagen coated 96 well plates (10 µg cm^−^²) in Williams E Medium (Biochrom, Germany) containing 2 mmol L^−1^ L-Anayl-L-Glutamine Dipeptide (GlutaMaxx, Thermo Scientific), 20 ng mL^−1^ (recombinant) epidermal growth factor (prospec Inc., USA), 7 ng mL^−1^ (recombinant) glucagon (prospec Inc., USA), 10 µg (2.5 U) mL^−1^ insulin from bovine pancreas (Sigma Aldrich, Germany), 5.5 µg mL^−1^ human holo-Transferrin (Sigma Aldrich, Germany), 6.7 ng mL^−1^ sodium selenite (Sigma Aldrich, Germany), 10 nmol mL^−1^ hydrocortisone (Sigma Aldrich, Germany),100 IU penicillin mL^−1^ and 100 IU mL^−1^ streptomycin (referred to WME^+^). After 6 h WME^+^ was replaced. After 16 h toxicity assays were performed for another 24 h as described below.

***In vitro*** **toxicity of polymethine dyes** were conducted to assess toxicity of DY-780 and DY-654 in HepG2, HEK293 and primary murine hepatocytes applying LDH (Roche, Germany) and Alamar blue assays (Life Technologies, Germany), respectively. Both cell lines were cultured in DMEM/F12 supplemented with 10% FCS and antibiotics (100 IU penicillin, 100 IU streptomycin) at 5% CO_2_ and humidity of 95% in an incubator (Heraeus Kendro HeraCell 150, USA). LDH release to the supernatant and alamar blue conversion were measured 24 h or 72 h after adding the indicated amounts of polymethine dyes according to assay kit protocols.

### Clinical Chemistry

Clinical biomarkers ASAT, ALAT, creatinine, urea, γ-GT and total bilirubin were measured using an Architect ci8200 integrated analysis system (Abbott GmbH & Co. KG, Germany).

**Quantification of bile acids** was achieved using a LC-MS/MS in-house assay assessing 15 bile acids in lithium-heparin plasma and from liver tissue lysates. After protein precipitation the filtration of plasma and homogenized liver samples were performed with Thomson Single Step Filter Vials (Thomson Instrument Company, California). Samples were loaded on an Agilent 1200 high performance liquid chromatography system (Agilent Technologies GmbH, Germany) with a CTC-PAL autosampler coupled to an API 4000 Triple Quadrupole mass spectrometer with electrospray ionization source (AB Sciex, Germany). All chromatographic separations were performed with a reverse-phase analytical column. The mobile phase consisted of water and methanol, both containing formic acid and ammonia acetate, at a total flow rate of 300 µL min^−1^.

### Peritoneal Contamination and Infection

Male FVB/NRj mice or male Wistar rats were injected intraperitoneally with a human stool suspension or 0.9% sterile saline solution for sham (both 1.25 µL g^−1^ BW). Mice or rats received fluid resuscitation (2.5 µL g^−1^ BW saline, s.c.) every 6 h. 16 h after infection dye clearance was measured applying different techniques as mentioned below.

### Liver ischemia-reperfusion injury

Male FVB/NRj mice or male Wistar rats were anesthetized and the abdomen opened with a vertical incision. The portal vein branches draining the left lateral lobe and median lobe were ligated for 45 min to induce a warm 70% liver ischemia. Afterwards the ligation was opened and skin layers were sutured up using a non-resorbable monofil 4–0 suture. After injection of 0.5% bupivacaine (7.5 µg BW, s.c.) at the site of surgery the narcosis was terminated and animals were kept for 24 h with food and water ad libitum.

### Kidney ischemia reperfusion injury

Male FVB/NRj mice or Wistar rats were anesthetized and the abdomen opened with a transverse incision of 1.5 cm beginning 0.5 cm below the xiphoid. Bilateral clamping of renal arteries was performed to induce a 100% renal ischemia for 45 min. Afterwards both clamps were removed, the abdomen closed, and bupivacain 0.5% (7.5 µg g^−1^ BW) was injected; animals were kept isolated for 24 h as described above.

### Glycerol induced acute renal injury

Male FVB/NRj mice or male Wistar rats were deprived of water for 16 h followed by several small intramuscular injections of a sterile 50% (v/v) glycerol solution in saline (10 µL g^−1^ BW) in the left and right hind limb under isoflurane anesthesia. Afterwards animals were kept isolated for 24 h as described above and water was offered again ad libitum.

### Plasma disappearance rate

To assess PDR in Wistar-rats catheters were placed in the right jugular vein, carotid artery and bile duct as described before^37^. DY-780 and DY-654 (140 pmol g^−1^ BW, each) were administered i.v. simultaneously. Bile was collected and 200 µL arterial blood taken into heparinized monovettes at indicated time points. To avoid collecting dead volume of the adapter and catheter, 100 µL of blood were aspirated prior to each sampling point and plasma was separated by centrifugation (2000 x g, 10 min). Urine was obtained before dye application and at the end of the sampling procedure. Polymethine dye concentrations in body fluids were measured via a spectrofluorometer as mentioned above.

### Intravital microscopy

Intravital microscopy allows insight into uptake and excretory procedures in the tissue *in vivo*. The left lateral liver lobe or left kidney was visualized in rats and mice. In rats the left jugular vein was cannulated to administer dyes; in mice tail-vein injection on the microscope was performed. Liver or kidneys were exposed via a right lateral abdominal incision. IVM in rats was performed using an inverted epifluorescence microscope (AxioObserver Z1; Carl Zeiss) as described previously, exploring NADPH auto-fluorescence to visualize tissue^20^. The inverted microscopy setup allows the flat and even exposure of the liver tissue to the cover glass, to obtain best possible constant focal volume between experiments. The procedure of exposure was performed as described in detail previously^20^. DY-654 or DY-780 were excited by a 100-Watt mercury lamp through multi-channel modules with two filter sets (for excitation and emission) (Table 1). Emitted light was captured on a high-resolution cooled, electron multiplying CCD camera (C9100-14, Hammamatsu Photonics, Japan). We focused on the brightest focal plane in each imaged area. Kinetics were calculated from at least 4 regions of interest imaged per animal and 4 animals per group. Data were normalized before averaging to the darkest 5% of the pixels in the image of the NAD(P)H channel (before injection of the dye).

**Table 1:** Filter sets used for epifluorescence intravital microscopy.

Fluorescent Molecule	Excitation	Emission	Exposure Time
NAD(P)H	365 to 395 nm	445 to 450 nm	50 ms
DY-654	630 to 640 nm	650 to 690 nm	150 ms
DY-780	775 to 805 nm	845 to 855 nm	150 ms

To obtain high-resolution images of DY-654 during kidney injury in mice an inverted laser-scanning microscope, LSM-780 (Carl Zeiss, Jena, Germany), equipped with an air corrected × 40, NA: 0.95) and water-oil emulsion × 63 (NA: 1.15) was used. DY-654 was excited using 633 nm laser and light was detected via a 650 nm long-pass filter on a GaAsP-Detector in similar fashion as described previously^37^.

### Polymer synthesis and characterization

The synthesis and characterization of poly(oligo(2-ethyl-2-oxazoline)methacrylate (POEtOxMA) comb-polymers is described in detail in the Supporting Information and Figure S4 and S5.

## Electronic supplementary material

Supplementary Material

Confocal intravital microscopy of murine kidney in control mice

Confocal intravital microscopy of murine kidney 24 h after glycerol-induced acute kidney injury

Confocal intravital microscopy of P1-DY-654 in the murine kidney: The dye-polymer conjugate P1-DY-654
