# Supplementary material for: Fast simultaneous assessment of renal and liver function using polymethine dyes in animal models of chronic and acute organ injury
Source: Sci Rep. 2017 Nov 13;7:15397. doi: 10.1038/s41598-017-14987-5 (PMC5684357; doi:10.1038/s41598-017-14987-5)
Supplement: Supplementary file 1 — Supplementary Material [file 41598_2017_14987_MOESM1_ESM.docx]

# Supplementary Material

# Fast simultaneous assessment of renal and liver function using polymethine dyes in animal models of chronic and acute organ injury

A. T. Press^1,2^, M. J. Butans^1,2^, T. P. Haider^3,4^, C. Weber^3,4^, S. Neugebauer^2,5^, M. Kiehntopf^2,5^, U. S. Schubert^2,3,4^, M. G. Clemens^6^, M. Bauer^1,2^, A. Kortgen^1,2,^*

**Affiliations**

*To whom correspondence should be addressed: andreas.kortgen@med.uni-jena.de

^1^ Department for Anesthesiology and Intensive Care Medicine, Jena University Hospital, Am Klinikum 1, 07747 Jena, Germany

^2^ Center for Sepsis and Control and Care, Jena University Hospital, Am Klinikum 1, 07747 Jena, Germany

^3^ Laboratory of Organic and Macromolecular Chemistry (IOMC), Friedrich Schiller University Jena, Humboldtstr. 10, 07743 Jena, Germany

^4^ Jena Center for Soft Matter (JCSM), Friedrich Schiller University Jena, Philosophenweg 7, 07743 Jena, Germany

^5^ Department for Clinical chemistry and Laboratory Medicine, Jena University Hospital, Am Klinikum 1, 07747 Jena, Germany

^6^ Department of Biological Sciences and Center for Biomedical Engineering and Science, University of North Carolina at Charlotte, 9201 University City Blvd, Charlotte NC 28223, USA

## Supplementary Methods: Polymer synthesis and characterization

**Materials**. All chemicals were purchased from standard suppliers and used without further purification, unless otherwise noted. The macromonomer oligo(2-ethyl-2-oxazoline)methacrylate (OEtOxMA) with an average degree of polymerization (DP) of 5 was synthesized as reported previously.^46^ 2,2’-Azobis(2-methylpropionitrile) (98%, Acros, AIBN) was recrystallized from methanol, and the chain transfer agent (CTA) 2-cyanopropyl dithiobenzoate (CPDB, 97%) was obtained from Strem. Preparative size exclusion chromatography was performed using BioBeads-SX1 and -SX3 from BioRad. Fluorescein-5-maleimide was purchased from TCI, and DY654-maleimide was a kind gift from Dyomics.

**Instrumentation**. ^1^H NMR spectra were recorded in CDCl_3_ on a Bruker Avance 300 MHz using the residual solvent resonance as an internal standard. Analytical ultracentrifugation (AUC) experiments were performed in water in a Beckman XLI analytical ultracentrifuge.^47^ UV-Vis absorption spectra were recorded in quartz cuvettes on a Specord 250 from Analytik Jena. UV-Vis emission spectra were recorded on a FP-6500 spectrofluorometer from Jasco. Labelling efficiencies were determined using UV-Vis absorption spectroscopy in phosphor buffered saline (PBS) at the absorption maximum of the respective dye and a calibration *via* dilution series using the free dyes. For characterization of OEtOxMA and monitoring of its polymerization, size exclusion chromatography (SEC) was measured on a Shimadzu system equipped with a SCL-10A system controller, a LC-10AD pump, a RID-10A refractive index detector (RID) and a SPD-10AV UV-vis detector using a solvent mixture containing chloroform, triethylamine, and isopropanol (94:4:2) at a flow rate of 1 mL min^-1^ on a PSS-SDV-linear M 5 μm column at 40 °C. The system was calibrated with linear polystyrene standards. For the SEC measurements with a diode array detector (DAD), an Agilent 1200 system was used. The system was equipped with both a PSS Gram30 and a PSS Gram1000 column in series, a G1310A pump, a G1362A RID as well as a G1315D DAD at 40 °C and operated at a flow rate of 1 mL min^-1^. *N,N*-Dimethylacetamide with 0.21% LiCl was used as eluent. The system was calibrated with linear polystyrene standards.

**Comb polymer synthesis (P1).** The synthesis of poly[oligo(2-ethyl-2-oxazoline)methacrylate)] (POEtOxMA, **P1**) by reversible addition fragmentation chain transfer (RAFT) polymerization of OEtOxMA was adopted from a method reported previously^22,46^: 2 g (3.38 mmol) OEtOxMA were weighed into a suitable reaction vessel. Corresponding to a ratio of [M]:[CTA]:[AIBN] of 200:1:0.25, 3.7 mg (0.017 mmol) CPDB and 0.7 mg (0.004 mmol) AIBN were added from suitable amounts of stock solutions in ethanol. Subsequently, the solution was diluted with ethanol to reach a final volume of 3.4 mL (corresponding to an initial monomer concentration [M]_0_ of 1 mol L^-1^). The vial was capped and the solution was gently purged with argon for 30 min to remove oxygen from the solution. Subsequently, the vial was immersed into an oil-bath pre-heated to 70 °C. After a polymerization time of 65 h, the polymerization was stopped by cooling and exposing the reaction mixture to air. Subsequent to determination of the monomer conversion, the volatiles were removed under reduced pressure and the residue was re-dissolved in tetrahydrofuran (THF). Residual OEtOxMA was removed by column chromatography on a BioBeads-SX1 column using THF as eluent. Subsequent to concentration of the desired fractions under reduced pressure, the purified comb polymer **P1** was precipitated into cold diethyl ether and dried under reduced pressure. The purity of **P1** was confirmed by means of ^1^H NMR spectroscopy, and the molar mass was determined by SEC as a relative and AUC as an absolute method.

Monomer conversion: 68%, M_n,theo_ = [M]:[CTA]·convsn.·600 g mol^-1^ = 82 000 g mol^-1^; M_n_(SEC) = 31 100 g mol^-1^; Đ_SEC_ = 1.20; M_AUC_ = 50 600 g mol^-1^(corresponding to a degree of polymerization of DP = M_AUC_ : 600 g mol^-1^ = 84).

**General procedure for conjugation of P1 with a maleimide-functional dye.** The conjugation procedure followed an adopted synthesis protocol for labelling of polymers obtained *via* RAFT polymerization with an acrylamide-functional dye recently published in our laboratories.^48^ It is based on the aminolysis of the dithiobenzoate end group of **P1** and an *in situ* 1,4-addition of the formed thiol to the maleimide-functional dye acting as a Michael-acceptor: 100 mg (0.002 mmol based on M_AUC_) **P1** were dissolved in 0.3 mL *N,N*-dimethylformamide (DMF), and appropriate amounts of dimethylphenylphosphine (DMPP, 0.6 mg; 0.0045 mol) and maleimide-functional dye (0.0015 mmol) were added from stock solutions in DMF. Subsequent to removal of oxygen from the solution, 1 mg (0.01 mmol) hexylamine in DMF (oxygen free) were added to promote cleavage of the dithiobenzoate moiety. The reaction was allowed to proceed for several days at room temperature, diluted with chloroform and washed several times with saturated aqueous sodium bicarbonate solution and brine to remove unreacted dye. Subsequent to drying of the organic phase with sodium sulfate, the volatiles were removed under reduced pressure. The residue was dissolved in THF and purified *via* column chromatography on a BioBeads SX-3 column (eluent THF). Finally, the dye-polymer conjugates were precipitated from cold diethyl ether and dried und reduced pressure. The covalent attachment of the dyes was confirmed *via* SEC with a DAD. Prior to determination of the labelling efficiency, the absence of residual solvents was assured by ^1^H NMR spectroscopy. It should be noted that all polymer-dye conjugates contain either one or none label per polymer chain, while the possibility of several dye molecules attached to the same polymer chain is excluded by the choice of the synthetic pathway. The polymer chains without dye will simply not be visible during intravital microscopy.

**P1-Flc** was synthesized according to the general procedure. 0.7 mg Fluorescein-5-maleimide were used. Reaction time 20 h; labelling efficiency: 12mol%. M_n_(SEC) = 32 400 g mol^-1^; Đ_SEC_ = 1.16.

**P1-DY654** was synthesized according to the general procedure. 1.7 mg DY654-maleimide was used. Reaction time 10 d; labelling efficiency: 1mol%. M_n_(SEC) = 31 400 g mol^-1^; Đ_SEC_ = 1.18.

###
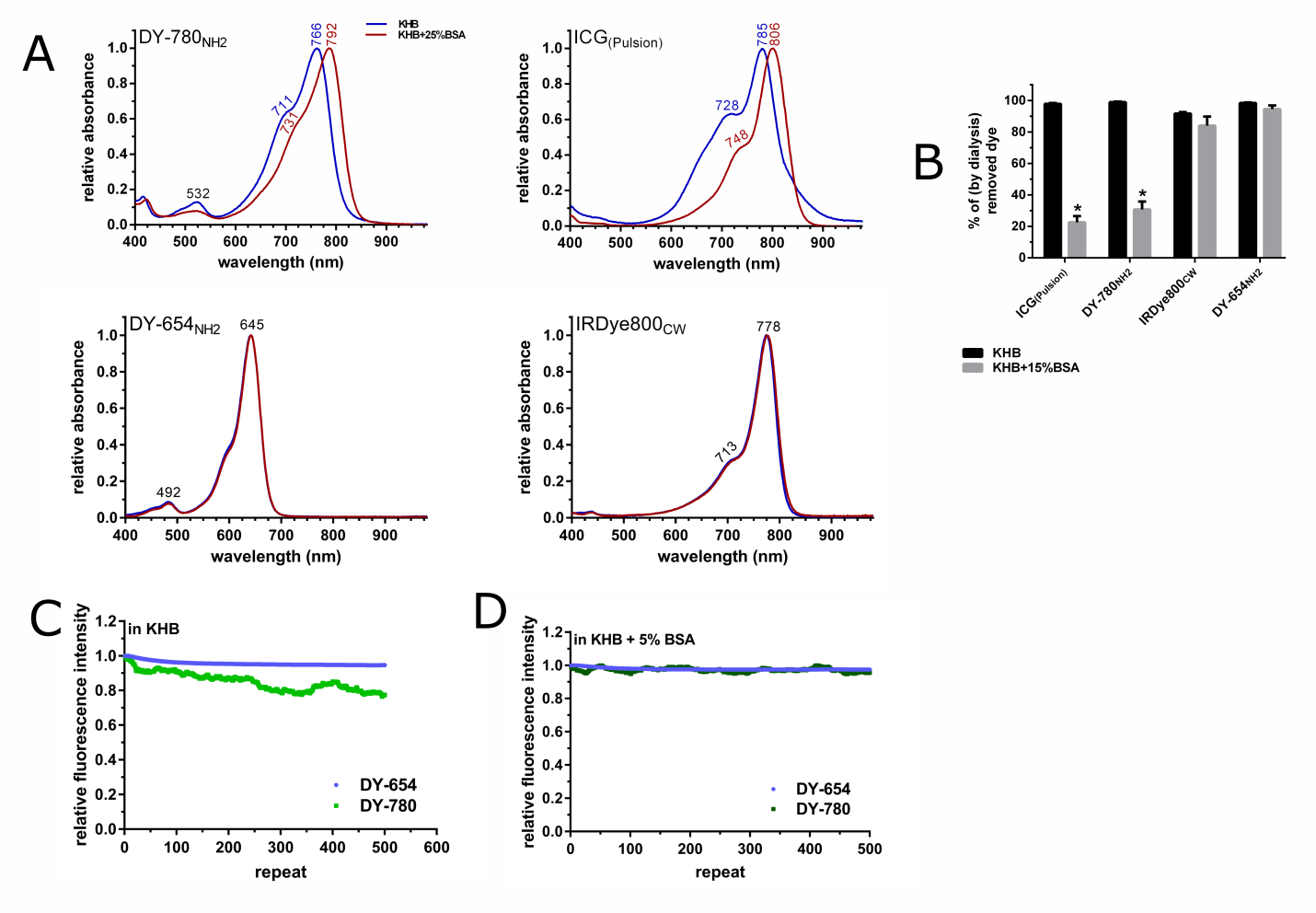


### Supplementary Figure 1: Albumin binding of hepatic and renal eliminated polymethine dyes

(**A**) Spectra of the hepatic eliminated dyes DY-780 and Indocyanine Green (ICG) as well as renal filtered dyes DY-654 and IRDye800cw diluted to 1 µmol L^-1^ in Krebs Henseleit Buffer (KHB) in the absence (blue line) and presence (red line) of 25% bovine serum albumin fraction V (BSA). Numbers label local peak absorption wavelength or the center of the spectral shoulder in nm. (**B**) Amount remaining dye after dialyzing 1 mL of different dye solutions (5 µmol L^-1^ in KHB) in the presence of 0 or 15% BSA for 8 h against 200 mL KHB twice using tubing with a molecular weight cut off of 6000 to 8000 Da. Bars represent the mean + SD from 6 replicates. Asterisks show to significance between KHB and respective KHB+15%BSA group (α<0.05, Mann-Whitney Test). (**C,D**) Photobleaching of DY-654 and DY-780 within 500 cycles of 100 flashes exposure on a multiplate reader (EnSpire, PerkinElmer) in KHB and KHB containing 5% BSA at 37 °C. Signal change was calculated from a linear regression and is given as fraction from the first measurement (in %) per 100 cycles: (**C**) -0.7% for DY-654, -3.7% for DY-780 (**D**) ‑0.09% for DY-654 and -0.18% for DY-780

###
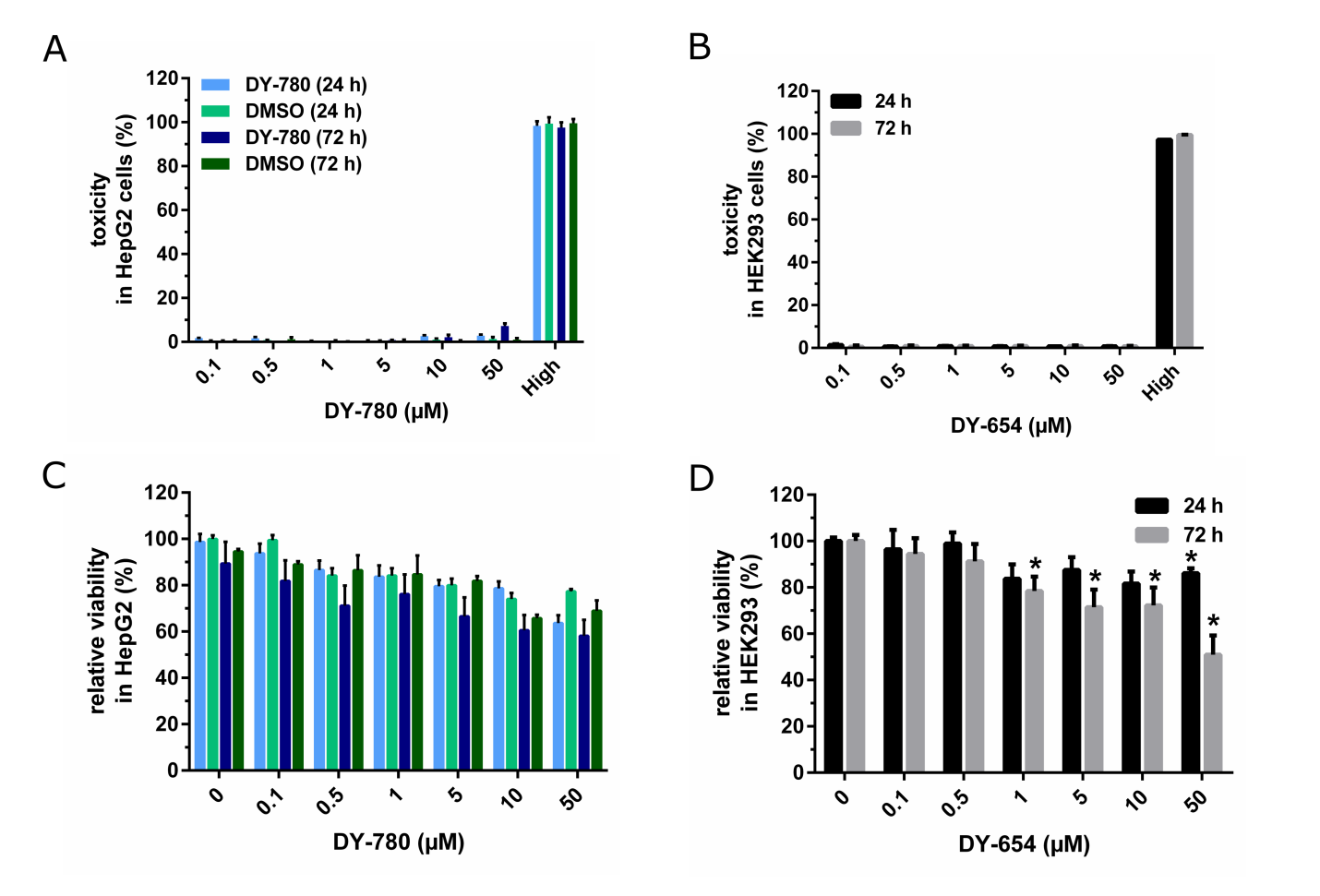
Supplementary Figure 2: Toxicity of polymethin dyes in HepG2 and HEK293 cells

(**A**) Toxicity (LDH-assay) after 24 h or 72 h on HepG2 cells for DY-780 or (**B**) HEK293 cells for DY-654 was assessed showing no cellular toxicity. (**A,B**) LDH release is expressed in % to completely lysed cells (High) by Triton-X solution. (**C**) The alamar blue assay revealed no significant decrease in cell viability of HepG2 cells after incubating those 24 h or 72 h with DY-780 compared to DMSO-vehicle control. (**D**) DY-654 incubation of HEK293 in contrast lead to a decreased cell viability at 24 h for c(DY-654) ≥ 50 µM and 72 h for c(DY-654) ≥ 1 μM. * depicts significance α < 0.05, Kruskal-Wallis-Anova compared to (**C**) DMSO-vehicle control or (**D**) media control of 4 independent experiments per group.


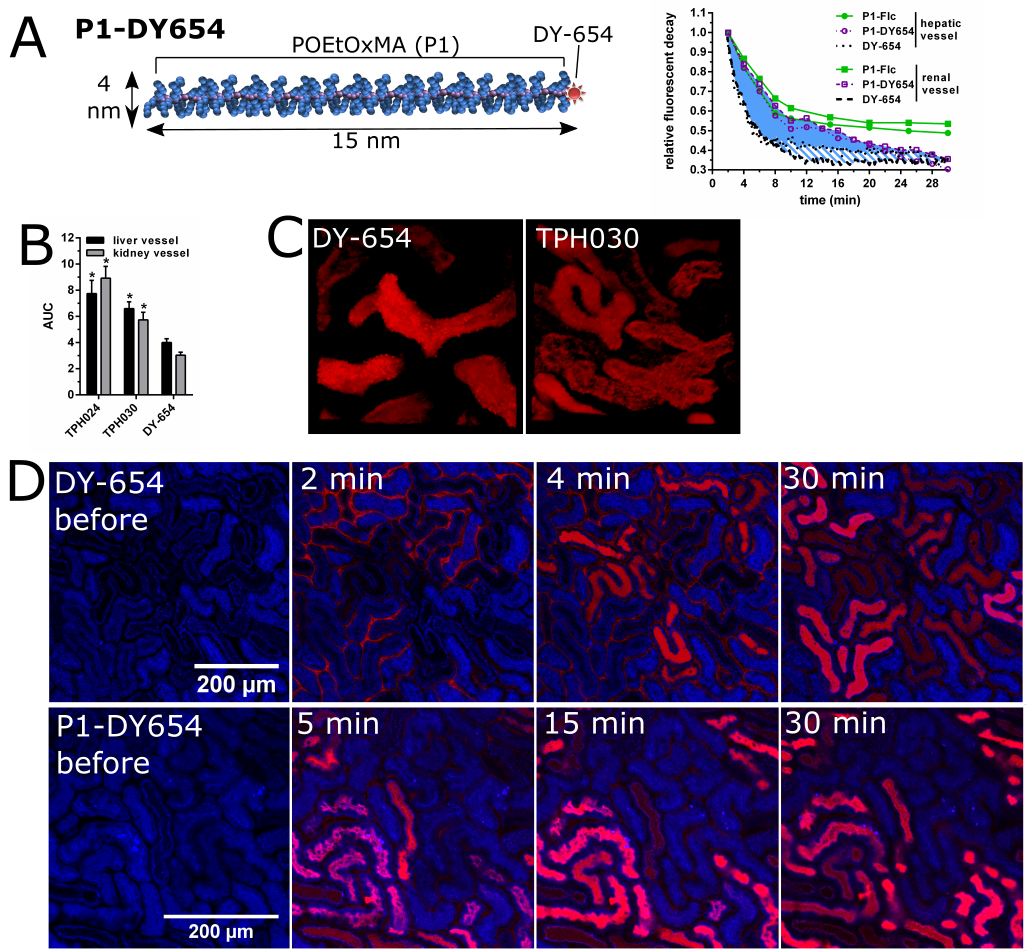


### Supplementary Figure 3: Elimination of comb polymer-dye conjugates

(**A**) Sterically inhibited polymers were synthesized exposing one dye molecule at the end such that all parts of the dye are available for interaction with the cells. Fluorescence decay in blood vessels of liver and kidney of DY-654 and polymers with covalently bound DY-654 (P1-DY654) or fluorescein (P1-Fic=TPH024), respectively. The analyses were based on 30 min time-series acquired by simultaneous confocal intravital microscopy of murine kidney and liver. (**B**) Area under the curve (AUC) analysis of kinetics shown in (A) (blue). (**C**) 3D-reconstruction of Z-Stacks from living animals treated with DY-654 for 45 min or P1-DY654 (=TPH030) for 90 min showing different localization in the tubular system of the murine kidney. (**D**) Image series of DY-654 (red, upper panel) and P1-DY654 (red, lower panel) and the tissue background visualized by the NAD(P)H auto fluorescence (blue) at indicated time points.


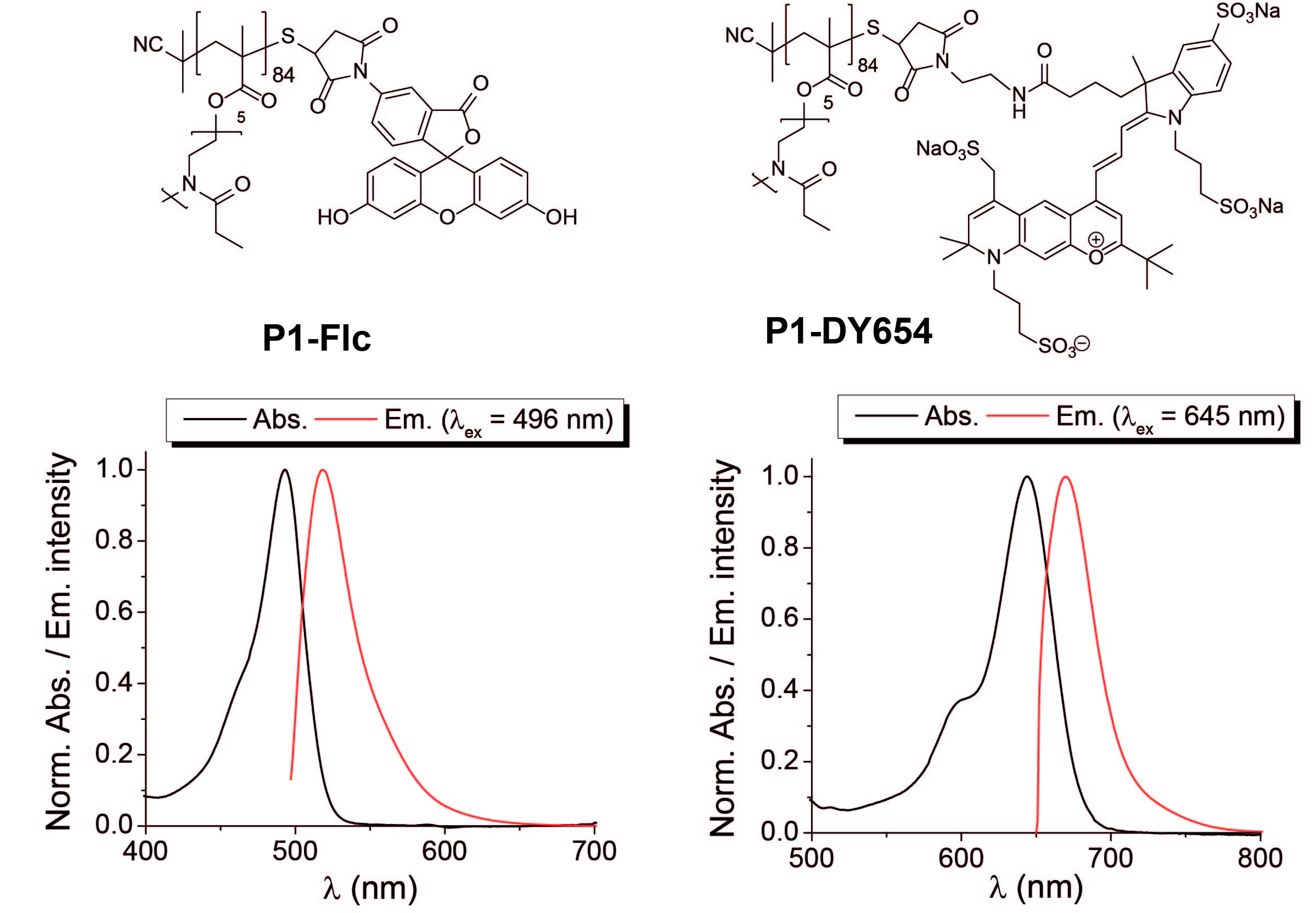


### Supplementary Figure 4: Investigation of the polymers’ fluorescent properties

Full schematic representation of the molecular structures of both polymer-dye conjugates **P1-Flc** (left) and **P1-DY654** (right) and normalized UV-Vis absorption and emission spectra of in PBS. For the emission spectra, the respective absorption maxima of the conjugated dyes were excited (λ_ex_ = 496 nm for **P1-Flc**, and λ_ex_ = 645 nm for **P1-DY654**).


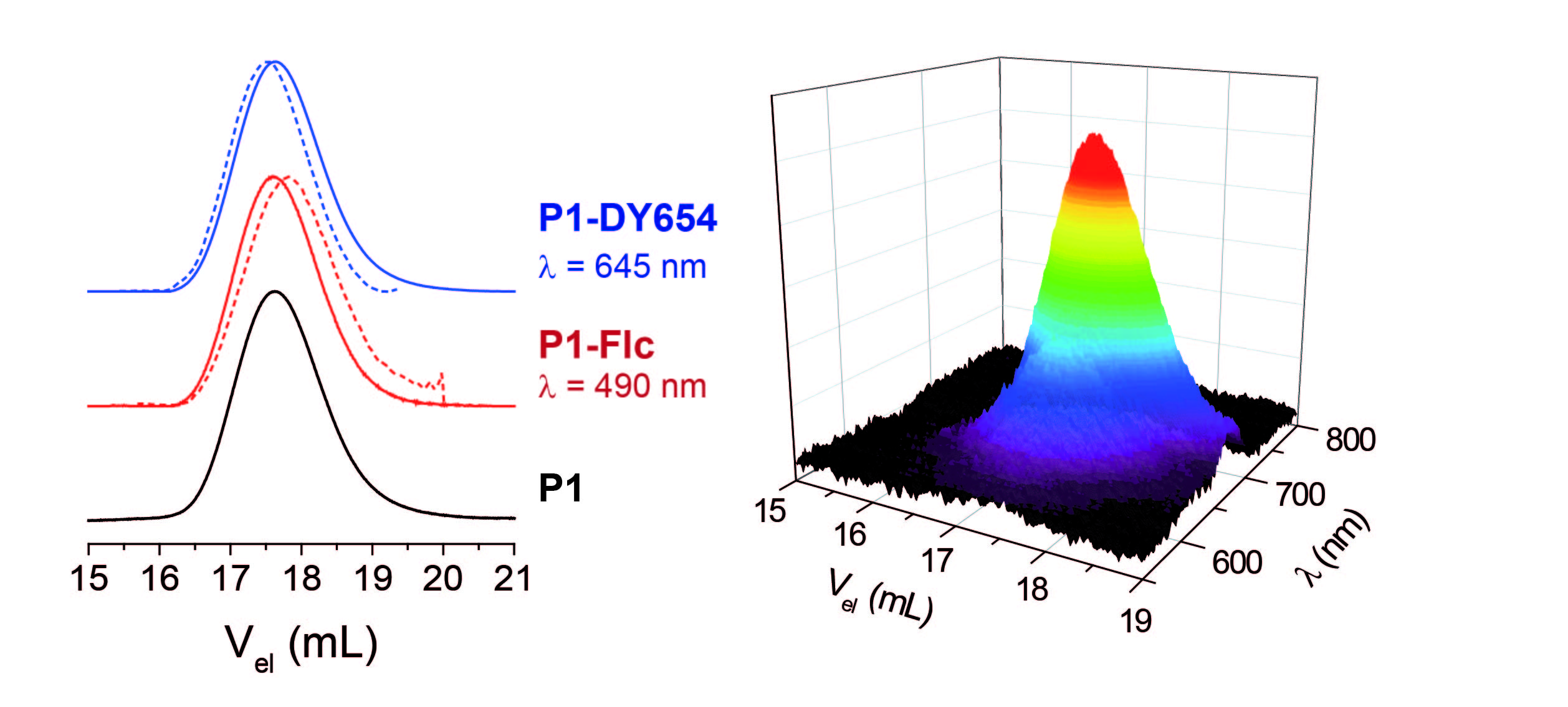


### Supplementary Figure 5: Characterization of the polymers by size exclusion chromatography

SEC analysis of the comb polymers (Eluent DMAc). **Left**: Overlay of the chromatograms of all polymers. The overlapping RID signals (solid lines) of all three polymers show that the comb polymer did not degrade upon conjugation with the dyes. The dashed lines depict the DAD signals recorded at the wavelength of the maximum absorbance of the conjugated dyes confirming the covalent attachment of the dyes at the polymer. **Right**: 3D-Plot obtained from SEC measurement of **P1-DY654** while recording the full absorption spectrum of the eluate using the DAD.

Supplementary Scheme 1: Polymers ynthesis

Schematic representation of the synthesis of POEtOxMA and the subsequent conjugation with maleimide functional dyes.

### Supplementary Video 1 Confocal intravital microscopy of murine kidney in control mice: DY-654 was administered i.v. *via* the tail-vein during image acquisition (6 frames per minute) showing different tubular segments subsequently appearing DY-654 positive. DY-654 is depicted in red. NAD(P)H autofluorescence is exploited to visualize cellular background (blue).

### Supplementary Video 2 Confocal intravital microscopy of murine kidney 24 h after glycerol-induced acute kidney injury: DY-654 was administered i.v. *via* the tailvein during image acquisition (6 frames per minute) showing different tubular segments subsequently appearing DY-654 positive. NAD(P)H autofluorescence is exploited to visualize cellular background (blue).

### Supplementary Video 3 Confocal intravital microscopy of P1-DY-654 in the murine kidney: The dye-polymer conjugate P1-DY-654 (red) was administered i.v. *via* the tail-vein during image acquisition (1 frame per minute) depicting the long circulation time over approximately 30 min compared to the free dye. Also the dye-conjugate appears in different tubular section. However, it is not secreted in the same speed as the free dye. NAD(P)H autofluorescence is exploited to visualize cellular background (blue).
